# The Performance of Five Bioelectrical Impedance Analysis Prediction Equations against Dual X-ray Absorptiometry in Estimating Appendicular Skeletal Muscle Mass in an Adult Australian Population

**DOI:** 10.3390/nu8040189

**Published:** 2016-03-29

**Authors:** Solomon C. Y. Yu, Alice Powell, Kareeann S. F. Khow, Renuka Visvanathan

**Affiliations:** 1 NHMRC Centre of Research Excellence: Frailty Trans-disciplinary Research to Achieve Healthy Ageing, University of Adelaide, Adelaide, SA 5000, Australia; kareeann.khow@adelaide.edu.au (K.S.F.K.); renuka.visvanathan@adelaide.edu.au (R.V.); 2 Adelaide Geriatrics Training and Research with Aged Care (G-TRAC) Center, School of Medicine, Faculty of Health Science, University of Adelaide, Adelaide, SA 5000, Australia; 3 Aged and Extended Care Service, The Queen Elizabeth Hospital, Central Adelaide Local Health Network, South Australia, SA 5000, Australia; alice.powell@sa.gov.au

**Keywords:** sarcopenia, bioelectrical impedance, muscle, dual-energy X-ray absorptiometry

## Abstract

Appendicular skeletal muscle mass (ASM) is a diagnostic criterion for sarcopenia. Bioelectrical impedance analysis (BIA) offers a bedside approach to measure ASM but the performance of BIA prediction equations (PE) varies with ethnicities and body composition. We aim to validate the performance of five PEs in estimating ASM against estimation by dual-energy X-ray absorptiometry (DXA). We recruited 195 healthy adult Australians and ASM was measured using single-frequency BIA. Bland-Altman analysis was used to assess the predictive accuracy of ASM as determined by BIA against DXA. Precision (root mean square error (RMSE)) and bias (mean error (ME)) were calculated according to the method of Sheiner and Beal. Four PEs (except that by Kim) showed ASM values that correlated strongly with ASM_DXA_ (*r* ranging from 0.96 to 0.97, *p* < 0.001). The Sergi equation performed the best with the lowest ME of −1.09 kg (CI: −0.84–−1.34, *p* < 0.001) and the RMSE was 2.09 kg (CI: 1.72–2.47). In men, the Kyle equation performed better with the lowest ME (−0.32 kg (CI: −0.66–0.02) and RMSE (1.54 kg (CI: 1.14–1.93)). The Sergi equation is applicable in adult Australians (Caucasian) whereas the Kyle equation can be considered in males. The need remains to validate PEs in other ethnicities and to develop equations suitable for multi-frequency BIA.

## 1. Introduction

Ageing is often associated with a progressive decline in both weight and skeletal muscle mass and an increase in body fat [1]. Sarcopenia, derived from the Greek word “sarx” meaning flesh and “penia” meaning loss, is the term that has been used to describe this age-related change. The prevalence of sarcopenia in those aged 65 years and over in Australia is estimated to be between 5% and 8%, whilst the condition affects one in five individuals aged 80 years and over [2].

Sarcopenia is associated with multiple adverse clinical outcomes, including disability, loss of independence, reduced quality of life, increased risk of falls, and the increased likelihood of death [3,4]. As a result of these adverse clinical outcomes, sarcopenia has become costly to the health system. It has been estimated, for example, that the direct healthcare cost attributable to sarcopenia in the US in 2000 was almost US $18.5 billion [2].

Not everyone who ages will succumb to sarcopenia, but everyone who succumbs will suffer a debilitating and costly decline. In an ageing population, this health issue is only going to become more pervasive, and diagnosis in clinical practice is essential for successful prevention and treatment [3]. Debate continues, however, about the exact definition of sarcopenia to take into clinical practice, and there have been six different recommendations to date [3]. Despite some variations between the recommendations, a consistent feature with all six is the consensus that appendicular skeletal muscle mass (ASM) is an important clinical parameter necessary for the diagnosis of sarcopenia [4]. Sarcopenia is asymptomatic at the early stages, when interventions such as exercise and appropriate protein supplementation could prevent or ameliorate adverse outcomes later in the individual’s life. If there were clinical tools available, on the other hand, that could assist the early identification of sarcopenia, intervention could be initiated early enough to make a significant difference to the person’s life as they age.

Although a wide range of techniques is available for clinicians to assess ASM, computed tomography (CT) and magnetic resonance imaging (MRI) are considered to be the most accurate for estimating muscle mass, given their ability to differentiate fat from other soft tissues of the body with greater precision [1]. However, both CT and MRI involve expensive equipment and exposure to radiation. Dual energy X-ray absorptiometry (DXA) is used more commonly in both research and clinical practice, but like CT and MRI, DXA involves equipment that is not portable and therefore not implementable in general practice (GP) clinic rooms or at the bedside. For many frail or homebound older people, none of these three imaging techniques is practical. Bioelectrical impedance analysis (BIA) is a non-invasive method to assess body composition that is portable, easy to use, relatively low-cost and free from radiation, and this method offers a solution for the measurement of body composition that is more convenient for the patient (e.g., in GP rooms).

Five prediction equations (PEs) that enable BIA estimation of ASM have been published (Table 1). These equations are yet to be validated for use in Australians. It is therefore unclear as to which equation should be implemented from a clinical and research perspective. The aim of this study was to compare the accuracy of selected published prediction equations for bioelectrical impedance analysis against dual energy X-ray absorptiometry-derived appendicular skeletal muscle mass.

## 2. Methods and Materials

### 2.1. Participants

In this study, 195 healthy subjects (age 18 to 83 years) were recruited from the western suburbs of Adelaide, South Australia, which are largely populated by residents of European decent. The study methodology is reported in greater detail elsewhere [10]. In brief, participants were randomly selected from the electronic white pages. Selected households were sent a letter of invitation and a brochure about the study. The person in the household aged 18 years or over who had recently had a birthday was eligible to participate in a brief telephone interview. A minimum of six telephone calls was made to each household before an individual was deemed non-contactable. Participants aged 18 years and over whose weight had been stable over the preceding three months were included. Those with the following conditions were excluded from the study: serious medical illness, known inflammatory disease, an acute illness in the three months before or in the two weeks following blood sampling, inability to hold morning medications and pregnancy. Participants were asked to refrain from smoking, alcohol consumption or vigorous exercise in the 24 h before their clinic assessments. Informed consent was obtained from all selected participants and the Central Northern Adelaide Health Service Ethics of Human Research Committee provided ethics approval for the study.

### 2.2. Anthropometry

On arrival for clinical testing, participants were asked to empty their bladders, following which their height and weight were measured. Height (m) was measured to the nearest 0.1 cm with shoes off using a wall-mounted stadiometer. Weight (kg) was measured to the nearest 0.1 kg with the subject wearing light clothing. Body mass index (BMI) was calculated as weight (kg) divided by height (m) square.

### 2.3. Bioelectrical Impedance Analysis (BIA)

BIA was assessed using the single frequency Quantum II Body Composition Analyzer (RJL Systems, Clinton Township, MI, USA) to estimate ASM. This device produces a 425 µA constant sinusoidal current at a single frequency of 50 kHz +/− 1%. Subjects were asked to remove their shoes, socks and jewellery. BIA measurements were performed with subjects in a supine position, with their arms positioned by their sides and palms facing down on a non-conductive examination chair. Two electrodes were placed on the right wrist and two others on the right ankle. On the wrist, one electrode was placed on the dorsal aspect next to the ulnar head and another was placed on the dorsal surface of the first joint of the middle finger. On the ankle, one electrode was placed on an imaginary line bisecting the medial malleolus and the other electrode on the base of the second toe. The principle applied was that impedance to the electrical flow of an injected current is related to the volume of the conductor (human body) and the square of the length of the conductor (height). The machine reported resistance and reactance values. These values were then entered into the PEs (Table 1) and the ASM was estimated.

### 2.4. Dual Energy X-ray Absorptiometry (DXA)

A Lunar PRODIGY whole-body scanner (GE Medical Systems, Madison, WI, USA), in conjunction with Encore 2002 software (GE Healthcare, Waukesha, WI, USA), was used to estimate ASM. The majority of subjects underwent DXA assessment on the same day as their BIA assessment, usually within two hours of arriving for their clinical assessment. All subjects had DXAs completed within two weeks. Given that subjects were healthy, body composition is unlikely to have changed significantly within that short period of time. Subjects were exposed to a radiation dose of approximately 2 µSv per scan and the scan took approximately five minutes to complete. For the DXA, subjects were required to lie supine on the scanning bed, wear light indoor clothing and remove any metal objects.

### 2.5. Statistical Analysis

Demographic characteristics are expressed as mean ± standard deviation. Independent sample *t*-testing was used to compare the means. Categorical variables were presented as frequencies and compared using Chi square analysis. Mean values of ASM were compared using the paired student *t*-test. The level of agreement between BIA and DXA was assessed using Bland Altman Analysis [11]. Precision (root mean square error (RMSE)) and bias (mean error (ME)) were calculated according to the method of Sheiner and Beal [12]. SPSS 19 for Windows (SPSS, Inc., Chicago, IL, USA) was used to perform the statistical analysis. A *p*-value of < 0.05 was considered to be statistically significant in this investigation.

## 3. Results

A total of 195 healthy adults aged between 18 and 83 years were recruited. The baseline demographic and health characteristics of the study population are summarised in Table 2. The women in this study were significantly older than the men (52.7 ± 14.7 *vs.* 48.0 ± 17.0 years, *p* = 0.04). There were more current smokers (13 ± 16.7 *vs.* 8 ± 6.8, *p* = 0.036) and people with hypertension (24 ± 30.8 *vs.* 20 ± 17.1, *p* = 0.035) in the male cohort when compared to the females. The low number of prescribed medications was in keeping with the fact that this was a healthy cohort. The men were taller (1.8 ± 0.1 *vs.* 1.6 ± 0.1 m, *p* < 0.001) and heavier (85.3 ± 16.0 *vs.* 70.6 ± 16.7 kg, *p* < 0.001) than the women.

Table 3 compares the performance of various BIA-derived ASM (ASM_PEs_) against the DXA-derived ASM (ASM_DXA_) in the total population, as well as the two gender groups. Except for the Kim equation (*r* = 0.44), there was strong correlation (*r* = 0.96 or 0.97) between the ASM_PEs_ and ASM_DXA_. When considering the total study cohort, the Sergi and Peniche equations underestimated ASM_DXA_, whereas all other equations overestimated ASM_DXA_. The Sergi equation was the best performer when the cohort was investigated as a whole, delivering the lowest root mean square error (RMSE) whilst the Kim equation performed the worst (RMSE 2.09 *vs.* 2.70). In men, the Kyle equation demonstrated the lowest ME (−0.32 kg (CI: −0.66 to 0.02)) and RMSE (1.54 (CI: 1.14–1.93)), thus providing the best approximation of ASM_PEs_ compared to ASM_DXA_. In women, the Sergi equation performed the best with the lowest ME (−0.17 kg (CI: −0.39 to −0.54)) and RMSE (1.23 kg (CI: 0.91 to 1.55)).

Table 4 compares the performance of various BIA PEs across various BMI groups. It should be noted that the number for the BMI < 18.5 kg/m^2^ was small (*n* = 5) and limited the comparison of the performance of various BIA PEs.

Apart from the Kim equation, the Sergi PE was observed to have the lowest ME, suggesting that it was the least biased PE when compared with the others. The Sergi equation was noted to consistently underestimate the ASM_DXA_ across the four BMI groupings.

The RMSE values of BIA PEs by Kyle, Sergi and Yoshida were comparable across the normal weight (18.5–25 kg/m^2^), overweight (BMI ≥ 25 kg/m^2^), and obese (BMI ≥ 30 kg/m^2^) groups. The Sergi equations performed best in the overweight and obese groups, with the lowest ME. For a BMI of 25.00–29.99 kg/m^2^, the ME = −1.18 kg (CI: −1.62, −0.74), while for a BMI ≥ 30 kg/m^2^, the ME = −0.72 (CI: −1.30 to −0.15). In addition, for a BMI of 25.00–29.99 kg/m^2^, the RMSE = 2.20 (CI: 1.56 to 2.85), while for a BMI ≥ 30 kg/m^2^, the RMSE = 2.12 (CI: 1.40 to 2.84) when employing the Sergi PE. The Sergi equations also produced the smallest 95% limit of agreement ((−4.92, 2.56) kg for a BMI of 25.00–29.99 kg/m^2^; (−4.75, 3.31) kg for BMI ≥ 30 kg/m^2^).

## 4. Discussion

Given the rising prevalence and multiple deleterious consequences of sarcopenia, it is critical that clinicians have an available method to assess ASM that is easily implementable within general practice rooms or at the bedside in hospitals or nursing homes. The BIA is one such tool but its performance is highly dependent on the performance of the PEs used. This study confirms that in a cohort of healthy Australians, the Sergi equation should be the preferred choice of BIA PE. This equation demonstrated the greatest predictive accuracy in women and/or overweight (BMI ≥ 25 kg/m^2^) and obese (BMI ≥ 30 kg/m^2^) individuals. The Kyle equation, on the other hand, performed better than the Sergi equation for men.

It is well known that the performance of any equation is related to the nature of the population in which the equation is developed [13]. Therefore, it is not surprising that in this study of healthy Australians predominantly of Caucasian decent, both the Sergi and Kyle equations, both developed in Caucasian population cohorts, outperformed the Kim and Yoshida equations that had been developed in Oriental cohorts. The same phenomenon was also observed in relation to the BMI. Both the cohort in the present study (between 26.4 and 27.4 kg/m^2^) and the cohort used for the development of the Sergi (27.0 kg/m^2^) equation recorded similar mean BMIs, which were higher than those observed in the development cohort for the Kyle PE (range: 22.5–25.8 kg/m^2^). Furthermore, the proportion of women to men in the Sergi study (60% women) was very similar to the ratio among the recruits for our study, whereas the Kyle study contained only 40% women. It is likely that for these reasons, the Sergi equation performed better than the Kyle equation when the total population was considered, while the Kyle equation outperformed the Sergi equation in men.

More noticeable was that both the Sergi and Kyle equations were developed using single-frequency BIA machines [5,6] similar to the machine used in our study. In contrast, the equations by Kim and Yoshida were developed using multi-frequency machines [8,9]. The difference in machine type is another reason the Kim and Yoshida equations may not have performed as well as the Sergi and Kyle equations. There are currently no prediction equations for ASM that have been developed using multi-frequency BIA machines in Caucasian cohorts. It has been reported, however, that the multi-frequency BIA is able to estimate extracellular water with greater accuracy, an important feature as the ratio of intracellular to extracellular water changes with age [14,15]. Therefore, the multi-frequency BIA machine is the preferred method for estimating ASM [14]. Further research to develop and validate an equation for ASM using multi-frequency BIA in Caucasians is therefore a critical next step.

It should be noted, in concluding, that the present study had several important limitations. It was a small study and some sub-groups were poorly represented, thus limiting the accuracy of sub-analysis. For example, there were very few (*n* = 5) underweight (BMI < 18.5 kg/m^2^) individuals. Body composition does vary by age and gender. Hydration status also might be different across the age groups. However, sub-analysis by gender and age was not performed because of the small sample size. The study cohort was also very healthy since individuals with multiple chronic diseases or acute illnesses had been excluded during the recruitment process. The weight and the good health of the study participants limit the generalizability of the research findings. However, the performance of multiple equations was evaluated and this is a major strength of this study. The information gathered provides a guide for researchers and clinicians in relation to the preferred PE for use when estimating ASM in Australian Caucasians. Replicating similar studies with other ethnic groups is critical to confronting sarcopenia, however, given the multicultural nature of many countries.

## 5. Conclusions

BIA-derived PEs can be used to estimate ASM. The PE developed by Sergi appears to provide a reasonable estimate of ASM in Australians of Caucasian decent. Where greater accuracy is required, the Kyle equation can be used in men. Given the multicultural composition of the Australian population, it will be important to validate equations for use in other ethnic groups, in underweight groups (*i.e.*, BMI < 18 kg/m^2^) and for both single- as well as multi-frequency BIA machines. It is also important to validate equations for common chronic diseases/comorbidities in this group.

## Figures and Tables

**Table 1:** Developed and validated bioelectrical impedance prediction equations used in estimation of appendicular skeletal muscle mass.

	BIA PEs	Reference Standard	BIA Instrument
	**Single frequency BIA Instrument**		
Sergi *et al.* (Caucasian) [5]	ASM (kg) = −3.964 + (0.227 × RI) + (0.095 × weight) + (1.384 × sex (men = 1, women = 0)) + (0.064 × Xc)	DXA Whole body—fan beam densitometer (Hologic QDR Discovery A, Hologic Italy)	Whole body tetrapolar BIA; BIA 101 RJL system (single frequency 50 kHz)
Kyle *et al.* (Caucasian) [6]	ASM (kg) = −4.211 + (0.267 × Ht^2^/resistance) + (0.095 × weight) + (1.909 × sex (men = 1, women = 0)) + (−0.012 × age) + (0.058 × X_c_)	DXA whole body (Hologic QDR 4500A, Hologic Inc., Waltham, MA, USA)	Xitron 4000B (single frequency 50 kHz)
Rangel Peniche *et al.* (Mexico) [7]	ASM (kg) = −0.05376 + (0.2394 × Ht^2^/R) + (2.708 × sex (men = 1, women = 0)) + (0.065 × weight)	DXA whole body (Hologic Explorer, QDR-4500W, Hologic Inc, Waltham, MA, USA)	SF-BIA Quantum X (single frequency 50 kHz)
	**Multi-frequency BIA Instrument**		
Kim *et al.* (Korean) [8]	ASM (kg) = ((Ht^2^/R × 0.104) + (age × −0.050) + (gender (men = 1, women = 0) × 2.954) + (weight × 0.055)) + 5.663	DXA appendicular (Lunar Corporation, Madison, WI	Inbody 3.0, Biospace Co., Korea (multifrequency BIA)
Yoshida *et al.* (Japanese) [9]	Women: ASM (kg) = 0.221 × (impedance index) + 0.117 × (weight) + 0.881 Men: ASM (kg) = 0.197 × (impedance index) + 0.179 × (weight) − 0.019	DXA whole body (QDR-4500A; Hologic, Waltham, MA, USA)	Multifrequency BIA MC-980A; Tanita, Tokyo, Japan (multifrequency BIA)

BIA = Bioelectrical Impedance Analysis, PE = prediction equation, DXA = Dual energy X-ray absorptiometry, SF-BIA = single frequency Bioelectrical Impedance Analysis, R = resistance, RI = resistance index (resistance/height^2^), X_c_ = reactance, Ht = Height.

**Table 2:** General characteristics of the CASA population.

**Characteristics**	**Men, *n* = 78**	**Women, *n* = 117**	***p*-Value**
Age (SD), years	48.0 (17.0)	52.7 (14.7)	0.04
Current smoker, *n* (%)	13 (16.7)	8 (6.8)	0.036
Physical activity level, *n* (%):			0.966
Sedentary	14 (17.9)	22 (18.8)	
Low	32 (41.0)	51 (43.6)	
Moderate	39 (20.0)	16 (20.5)	
High	37 (19.0)	16 (20.5)	
**Health status/Chronic diseases**			
Diabetes mellitus, *n* (%)	3 (3.8)	3 (2.6)	0.686
Hypertension, *n* (%)	24 (30.8)	20 (17.1)	0.035
Hypercholesterolaemia, *n* (%)	30 (38.5)	51 (43.6)	0.554
Osteoarthritis, *n* (%)	7 (9)	14 (12)	0.639
IHD/angina, *n* (%)	0 (0)	0 (0)	NA
COPD, *n* (%)	1 (1.3)	0 (0)	NA
TIA, *n* (%)	1 (1.3)	0 (0)	NA
Past history cancer, *n* (%)			NA
Skin	1 (1.3)	2 (1.7)	
Breast	0 (0)	1 (0.9)	
No cancer	77 (98.7)	114 (97.4)	
Depression, *n* (%)	0 (0)	9 (7.7)	NA
**Medications**			
Number of prescribed meds, mean (SD)	0.7 (1.3)	1.4 (1.6)	0.003
Number of “over the counter” meds, mean (SD)	0.6 (1.1)	1.1 (2.0)	0.046
**Body composition**			
Weight, mean (SD), kg	85.3 (16.0)	70.6 (16.7)	<0.001
Height, mean (SD), m	1.8 (0.1)	1.6 (0.1)	<0.001
BMI, mean (SD), kg/m^2^	27.4 (4.5)	26.4 (5.7)	0.22
BMI categories:			
Underweight (<18.5), *n* (%)	1 (1.3)	4 (3.4)	NA
Normal (18.5–24.99), *n* (%)	23 (29.5)	47 (40.2)	
Overweight (25–30), *n* (%)	31 (39.7)	40 (34.2)	
Obese (≥30), *n* (%)	23 (29.5)	26 (22.2)	
ASMM_DXA_ (SD), kg	27.2 (4.5)	17.4 (3.2)	0.04
Resistance (SD), Ω	456.1 (47.9)	571.9 (76.9)	<0.001
Reactance (SD), Ω	54.3 (8.4)	56.9 (8.8)	0.04
Impedance (SD), Ω	459.4 (48.0)	574.8 (76.9)	<0.001

NA = not applicable, Ω = ohm, ASM = Appendicular Skeletal Muscle Mass.

**Table 3:** Validation of the five BIA prediction equations in the CASA study.

	Mean (SD), kg	Mean Error (95% CI), kg	*p*-Value for Mean Error	Pearsons *R*	95% Limits of Agreement	RMSE (95% CI)
**Total (*n* = 195)**						
ASM_DXA_	21.30 (6.11)					
ASM_Sergi_	20.21 (4.98)	−1.09 (−1.34, −0.84)	<0.001	0.97 *	−2.50, 4.68	2.09 (1.72, 2.47)
ASM_Kyle_	22.63 (5.13)	1.33 (1.06, 1.60)	<0.001	0.96 *	−5.17, 2.52	2.33 (1.61, 3.06)
ASM_Peniche_	19.48 (5.39)	−1.82 (−2.04, −1.60)	<0.001	0.97 *	−4.90, 1.26	2.38 (2.00, 2.77)
ASM_Kim_	22.99 (1.87)	1.69 (0.91, 2.47)	<0.001	0.44 *	−12.78, 9.40	5.78 (5.06. 6.51)
ASM_Yoshida_	23.29 (6.09)	1.99 (1.73, 2.25)	<0.001	0.96 *	>−5.65, 1.68	>2.70 (2.28, 3.12)
**Men (*n* = 78)**						
ASM_DXA_	27.20 (4.46)					
ASM_Sergi_	24.74 (3.68)	−2.47 (−2.84, −2.10)	<0.001	0.94 *	−0.80, 4.93	2.95 (2.28, 3.62)
ASM_Kyle_	26.89 (4.17)	−0.32 (−0.66, 0.02)	0.067	0.94 *	−2.71, 3.34	1.54 (1.14, 1.93)
ASM_Peniche_	24.80 (3.48)	−2.41 (−2.80, −2.01)	<0.001	0.93 *	−5.90, 1.08	2.96 (2.28, 3.64)
ASM_Kim_	22.91 (1.92)	−4.28 (−5.01, −3.56)	<0.001	0.77 *	−2.16, 10.73	5.35 (4.07, 6.63)
ASM_Yoshida_	28.81 (4.70)	1.61 (1.13, 2.08)	<0.001	0.89 *	>−5.85, 2.63	>2.65 (1.98, 3.32)
**Women (*n* = 117)**						
ASM_DXA_	17.37 (3.19)					
ASM_Sergi_	17.20 (3.10)	−0.17 (−0.39, −0.54)	0.135	0.93	−2.28, 2.62	1.23 (0.91, 1.55)
ASM_Kyle_	19.79 (3.48)	2.42 (2.19, 2.66)	<0.001	0.93	−4.98, 0.14	2.74 (2.25, 3.23)
ASM_Peniche_	15.94 (3.00)	−1.43 (−1.66, −1.20)	<0.001	0.92 *	−3.93, 1.07	1.90 (1.48, 2.32)
ASM_Kim_	23.04 (1.83)	5.67 (5.28, 6.06)	<0.001	0.77	−9.94, −1.41	6.06 (5.22, 6.90)
ASM_Yoshida_	19.61 (3.63)	2.24 (1.05, 2.53)	<0.001	0.90	−5.39, 0.90	2.74 (2.19, 3.28)

* *p* value < 0.001, RMSE = root mean square error.

**Table 4:** Comparison of the five BIA prediction equations across different body mass index (BMI) groupings.

	Mean (SD), kg	Mean Error (95% CI), kg	*p*-Value for Mean Error	Pearsons *R*	95% Limits of Agreement	RMSE (95% CI)
**BMI < 18.5 kg/m^2^, *n* = 5**						
ASM_DXA_	13.90 (3.43)					
ASM_Sergi_	12.56 (2.43)	−1.35 (−3.40, 0.70)	0.142	0.90 *	−4.65, 1.95	2.00 (−0.92, 4.91)
ASM_Kyle_	14.38 (2.32)	0.46 (−1.69, 2.64)	0.578	0.89 *	−3.02, 3.96	1.63 (−0.20, 3.46)
ASM_Peniche_	12.15 (2.75)	−1.75 (−3.19, −0.32)	0.028	0.95 *	−4.06, 0.56	2.03 (−0.82, 2.88)
ASM_Kim_	20.10 (1.72)	6.19 (0.60, 11.78)	0.037	0.47	−2.81, 15.19	7.39 (−0.16, 14.93)
ASM_Yoshida_	14.35 (2.27)	0.45 (−1.28, 2.17)	0.513	0.96 *	−2.32, 3.23	1.32 (0.29, 2.35)
**BMI 18.5–24.99 kg/m^2^, *n* = 70**						
ASM_DXA_	19.08 (4.90)					
ASM_Sergi_	17.84 (3.70)	−1.23 (−1.69, −0.86)	<0.001	0.97 *	−4.34, 1.88	1.98 (1.34, 2.62)
ASM_Kyle_	20.14 (3.69)	1.07 (0.67, 1.46)	<0.001	0.97 *	−2.23, 4.37	1.96 (1.50, 2.41)
ASM_Peniche_	17.10 (4.25)	−1.98 (−2.31, −1.65)	<0.001	0.97 *	−4.74, 0.79	2.40 (−1.02, 5.82)
ASM_Kim_	21.78 (1.18)	2.70 (1.51, 3.89)	<0.001	0.04	−7.29, 12.69	5.65 (4.46, 6.84)
ASM_Yoshida_	20.05 (4.15)	0.97 (0.63, 1.32)	<0.001	0.96 *	−3.83, 1.89	1.72 (1.31, 2.13)
**BMI 25.00–29.99 kg/m^2^, *n* = 71**						
ASM_DXA_	21.89 (6.32)					
ASM_Sergi_	20.70 (4.85)	−1.18 (−1.62, −0.74)	<0.001	0.98 *	−4.92, 2.56	2.20 (1.56, 2.85)
ASM_Kyle_	23.14 (4.93)	1.26 (0.79, 1.73)	<0.001	0.97 *	−2.69, 5.21	2.33 (1.78, 2.88)
ASM_Peniche_	20.02 (5.42)	−1.86 (−2.23, −1.49)	<0.001	0.98 *	−4.97, 1.25	2.42 (1.75, 3.09)
ASM_Kim_	23.06 (1.31)	1.18 (−0.20, 2.56)	0.09	0.47 *	−10.47, 12.83	5.90 (4.69, 7.12)
ASM_Yoshida_	23.87 (5.72)	2.00 (1.63, 2.35)	<0.001	0.97 *	−1.02, 5.02	2.49 (1.89, 3.09)
**BMI ≥ 30 kg/m^2^, *n* = 49**						
ASM_DXA_	24.39 (5.81)					
ASM_Sergi_	23.67 (4.37)	−0.72 (−1.30, −0.15)	0.015	0.96 *	−4.75, 3.31	2.12 (1.40, 2.84)
ASM_Kyle_	26.28 (4.54)	1.89 (1.27, 2.50)	<0.001	0.94 *	−2.40, 6.18	2.84 (1.95, 3.73)
ASM_Peniche_	22.84 (4.74)	−1.55 (−2.06, −1.05)	<0.001	0.97 *	−5.07, 1.97	2.33 (1.52, 3.14)
ASM_Kim_	24.91 (1.60)	0.52 (−1.10, 2.15)	0.520	0.24	−10.78, 11.82	5.62 (4.14, 7.10)
ASM_Yoshida_	28.00 (5.48)	3.60 (3.11, 4.07)	<0.001	0.96 *	0.26, 6.94	3.95 (2.96, 4.95)

ASM = appendicular skeletal muscle mass, RMSE = root mean square error.
